# Bovine lactoferricin exerts antibacterial activity against four Gram-negative pathogenic bacteria by transforming its molecular structure

**DOI:** 10.3389/fcimb.2025.1508895

**Published:** 2025-05-16

**Authors:** Jie Pei, Lin Xiong, Xiaoyun Wu, Min Chu, Pengjia Bao, Qianyun Ge, Xian Guo

**Affiliations:** ^1^ Key Laboratory of Yak Breeding in Gansu Province, Lanzhou Institute of Husbandry and Pharmaceutical Sciences, Chinese Academy of Agricultural Sciences, Lanzhou, Gansu, China; ^2^ Key Laboratory of Animal Genetics and Breeding on Tibetan Plateau, Ministry of Agriculture and Rural Affairs, Lanzhou, Gansu, China

**Keywords:** lactoferricin, antimicrobial peptide, disulfide bond, secondary structure, conformational transformation, antibacterial activity

## Abstract

The emergence and development of pathogenic bacterial resistance to antibiotics pose significant challenges to human health. Antimicrobial peptides (AMPs) are considered promising alternatives to conventional antibiotics. Lactoferricin (Lfcin), a cationic AMP located in the N-terminal region of lactoferrin, serves as the antimicrobial active center of the intact protein. The presence of two cysteines in Lfcin allows for the formation of an intramolecular disulfide bond, which may influence its molecular structure and antibacterial function. To investigate this hypothesis, we synthesized, purified, and identified bovine Lfcin along with two derivatives: Lfcin with a disulfide bond (Lfcin DB) and a mutated form that cannot form the disulfide bond (Lfcin C36G). We analyzed the circular dichroism spectra of these peptides under varying ionic and hydrophobic conditions, while their tertiary structures were predicted using AlphaFold3. Results indicated that increased ionic strength reduced the random coil ratios across all peptides. The secondary structure of Lfcin showed similar percentages with Lfcin C36G in the H_2_O and similar ratios with Lfcin DB under hydrophobic conditions. AlphaFold3-predicted models revealed two distinct structures: one predominantly adopting α-helix conformations and the other characterized by β-sheet topology. Furthermore, we evaluated the antibacterial activity of the peptides against four Gram-negative bacteria, including *Escherichia coli*, *Klebsiella pneumoniae*, *Pseudomonas aeruginosa*, and *Salmonella gallinarum*. The synthetic peptides demonstrated broad-spectrum antibacterial activity, with Lfcin exhibiting superior efficacy compared to its derivatives. Our findings suggest that Lfcin can reversibly interconvert between two distinct molecular states under varying ionic strengths and hydrophobic effects, with the resulting structural transformations enhancing its antibacterial function.

## 1 Introduction

Bacterial pathogens have long been a significant cause of human morbidity and mortality worldwide (Zhou et al., 2022). The discovery of antibiotics marked a transformative milestone in medicine, providing a rapid and effective means to combat bacterial infections, which substantially improved human health and dramatically extended life expectancy (Fischbach and Walsh, 2009; Cook and Wright, 2022). However, the escalating overuse and misuse of antibiotics have accelerated the emergence of antibiotic-resistant bacterial strains, diminishing the efficacy of major antibiotic classes (Vaughn et al., 2021; Amna et al., 2024). In 2019, the World Health Organization (WHO) estimated that antimicrobial resistance directly caused 1.27 million deaths and contributed to an additional 4.95 million fatalities worldwide (Harald, 2024). A notably alarming consequence of this trend is the emergence of multidrug-resistant (MDR) pathogenic bacteria, which evade the effects of multiple antibiotic families. Among these, the ESKAPE pathogens—*Enterococcus faecium*, *Staphylococcus aureus*, *Klebsiella pneumoniae* (*K. pneumoniae*), *Acinetobacter baumannii*, *Pseudomonas aeruginosa* (*P. aeruginosa*), and *Enterobacter* spp.—represent a critical group prioritized by the WHO as requiring urgent therapeutic solutions. Despite advances in antibiotic development, including next-generation β-lactamase inhibitors, treatment options for ESKAPE-related infections remain limited (Miller and Arias, 2024). This persistent challenge highlights the imperative for innovative strategies to address antibiotic resistance, emphasizing the need for novel antimicrobial agents and resistance-mitigation approaches (Miethke et al., 2021).

Antimicrobial peptides (AMPs) are a diverse group of naturally occurring molecules that play a crucial role in the innate immune system, serving as the first line of defense against a broad spectrum of pathogens (Wimley and Hristova, 2011; Fry, 2018). Unlike traditional antibiotics, AMPs employ unique mechanisms of action, such as disrupting bacterial membranes (Islam et al., 2023). AMPs can destabilize bacterial membrane integrity by leveraging their hydrophobic and cationic properties to interact with the negatively charged bacterial membranes via electrostatic attraction. This interaction leads to membrane disruption, leakage of cellular contents, and ultimately bacterial death (Melo et al., 2009). In addition to their membrane-targeting action, certain α-helical AMPs penetrate bacterial membranes to bind nucleic acids, thereby disrupting cellular metabolism and inducing rapid bactericidal effects (Vizioli and Salzet, 2002). Importantly, the rapid multifaceted mechanisms of AMPs make it difficult for bacteria to develop resistance through genetic mutations (Lei et al., 2019). The multifunctionality of AMPs, combined with their broad-spectrum activity and lower likelihood of resistance development, makes them promising candidates for the development of new antibacterial therapies in the fight against rising antibiotic resistance (Andersson et al., 2016; Lazzaro et al., 2020)​.

One notable AMP is bovine lactoferricin (Lfcin), a derivative of the milk protein lactoferrin (LF), which exhibits potent antibacterial activity against a broad range of pathogens (Gifford et al., 2005). LF is a natural glycoprotein first identified in cow’s milk and produced by mucosal epithelial cells and neutrophils (Hao et al., 2019; Wang et al., 2019). LF has several biological functions, including antimicrobial, antiviral, anti-inflammatory, anticarcinogenic, and immunoregulatory activities (Mayeur et al., 2016). Lfcin, a peptide released from the N-terminal region of LF through proteolysis, retains many functions of LF and, in certain cases, exhibits even greater activity (Wakabayashi et al., 2003; Gifford et al., 2005). For example, Lfcin demonstrates significantly enhanced antibacterial activity compared to LF and its other hydrolysates, suggesting that Lfcin may represent the antimicrobial core of LF (Bellamy et al., 1992a, 1992; Gifford et al., 2005). Bovine Lfcin consists of 25 amino acid residues, corresponding to positions 17–41 near the N-terminus of mature bovine LF, and contains eight positively charged and ten hydrophobic residues (Sinha et al., 2013). These residues impart amphipathic properties to the peptide, enabling its potent antibacterial action against a wide array of pathogenic bacteria (Wang et al., 2017; Sun et al., 2018; Vega Chaparro et al., 2018). Moreover, bovine Lfcin has been shown to possess stronger antibacterial activity compared to Lfcins from other mammals (Vorland et al., 1998; Rekdal et al., 1999). Given its unique molecular characteristics, bovine Lfcin serves as an excellent candidate for the development of therapeutic molecules through structure-based design strategies aimed at combating infections caused by various pathogens (Svendsen et al., 2019).

**Figure 1:**
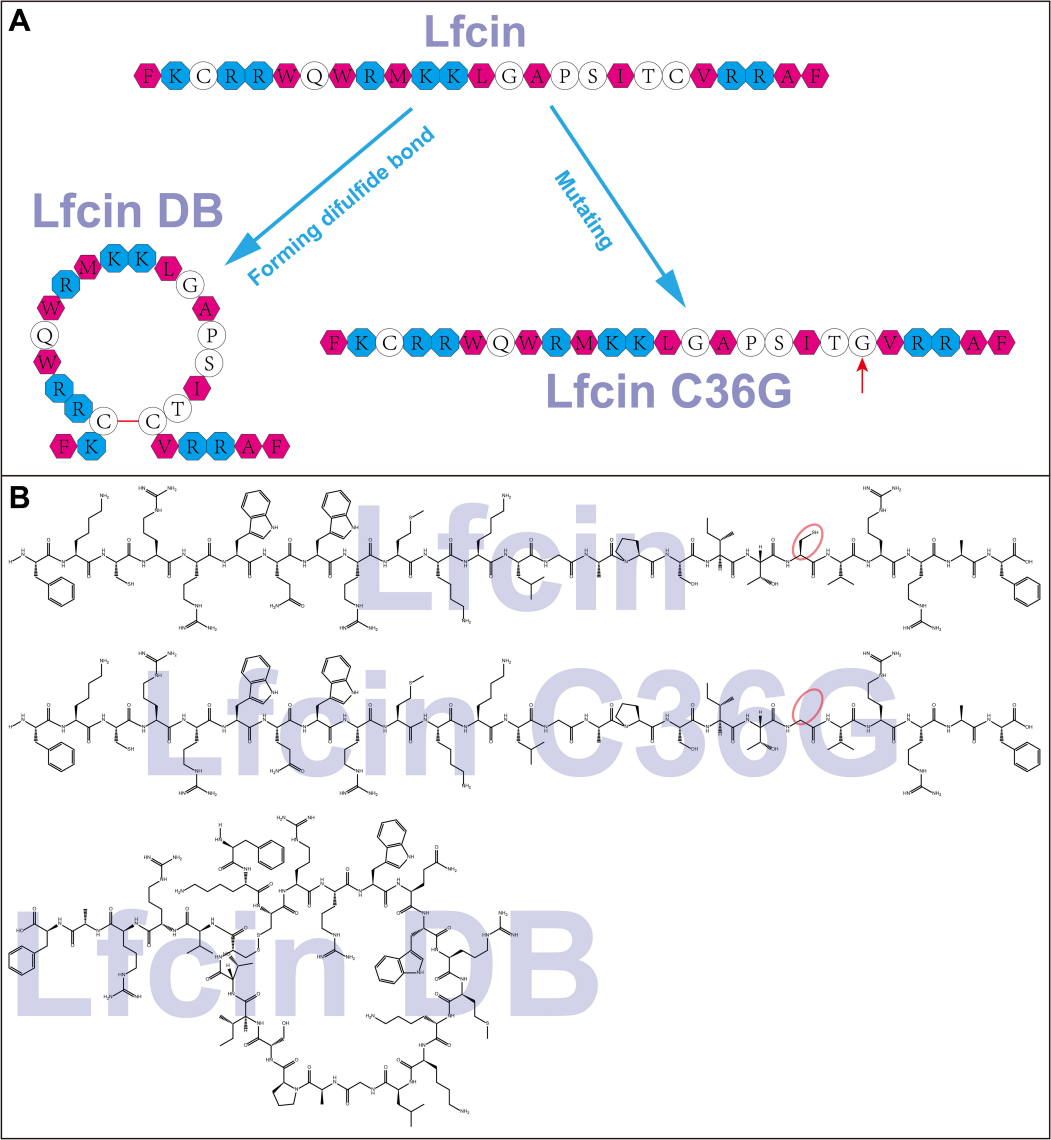
Schematic representation **(A)** and molecular structures **(B)** of Lfcin and its derivatives. Basic amino acids are represented by cyan octagons, while hydrophobic amino acids are shown as magenta hexagons. Lfcin DB is formed through a disulfide bond between C19 and C36, indicated by a red bar, resulting in a macrocyclic structure. Lfcin C36G arises from a mutation at position 36, where cysteine is replaced by glycine, illustrated by a red arrow. The red ellipses highlight the difference between the mercaptomethyl group in Lfcin and the hydrogen atom in Lfcin C36G.

One strategy to enhance the efficacy of AMPs involves introducing a disulfide bridge between two Cys residues to form a macrocyclic conformation, which can improve the peptide’s proteolytic stability, cell membrane permeability, and target binding affinity (Brown et al., 2024). The increased membrane permeability may facilitate more effective disruption of the plasma membrane, thereby enhancing antibacterial activity. Based on this, we hypothesize that bovine Lfcin could undergo structural changes through the formation or breakage of an intramolecular disulfide bond, potentially altering its antibacterial activity by modifying its interaction with bacterial membrane. To test this, we synthesized and characterized bovine Lfcin and its two Lfcin derivatives: Lfcin C36G, in which Cys36 is replaced with a Gly residue to prevent disulfide bond formation, and Lfcin BD, which forms a disulfide bond at Cys19 and Cys36. The preliminary structures of the peptides are shown in **Figure 1**. Circular dichroism (CD) spectra of the peptides were measured in solutions with varying ionic and hydrophobic conditions to analyze their secondary structures. Moreover, the antibacterial activities of these peptides were evaluated against four Gram-negative MDR bacteria including *Escherichia coli* (*E. coli*) (Dunn et al., 2019), *K. pneumoniae* (Wyres et al., 2020), *P. aeruginosa* (Qin et al., 2022), and *Salmonella gallinarum* (*S. gallinarum*) (Mouad et al., 2024). These pathogens represent a significant threat to global public health due to their resistance to multiple classes of antibiotics. This study aims to elucidate the disulfide-mediated conformational dynamics of Lfcin and its functional consequences, specifically investigating whether intramolecular disulfide bond rearrangement modulates structural plasticity to alter bactericidal efficacy against the above Gram-negative pathogens. This study contributes to a broader understanding of AMPs and their mechanisms of action, providing insights into the potential of LfcinB for developing new antimicrobial treatments.

## 2 Materials and methods

### 2.1 Reagents and chemicals

Mueller-Hinton agar (MHA), SPC agar, Mueller-Hinton broth (MHB), *E. coli* ATCC 25922, *K. pneumoniae* ATCC 4352, and *P. aeruginosa* ATCC 27853 were obtained from the American Type Culture Collection (ATCC, VA, USA). The bacterial strain *S. gallinarum* was isolated from liver tissues of clinically infected chickens and identified through biochemical characterization, serotyping, and molecular confirmation (Sannat et al., 2017; Lijuan et al., 2017). 1,2-ethanedithiol (EDT), 4-methylpiperidine, pyridine, N,N-diisopropylethylamine (DIPEA), ninhydrin, and triisopropylsilane (TIPS) were sourced from Sigma-Aldrich (MO, USA). Fmoc-amino acids, N,N-dicyclohexylcarbodiimide (DCC), rink amide resin, and 6-chloro-1-hydroxy-benzotriazole (6-Cl-HOBt) were purchased from AAPPTec (KY, USA). Absolute ethanol, acetonitrile (ACN), dichloromethane (DCM), diethyl ether, isopropylalcohol (IPA), methanol, N,N-dimethylformamide (DMF), and trifluoroacetic acid (TFA) were sourced from Honeywell-Burdick & Jackson (MI, USA). All reagents were used as received without further purification.

### 2.2 Peptide synthesis

The peptides were synthesized by manually using solid-phase peptide synthesis (SPPS) following the Fmoc/tBu strategy (Remuzgo et al., 2014). Briefly, Rink amide resin (0.46 meq/g) was used as the solid support. (a) The resin was treated with 20% 4-methylpiperidine in DMF to remove the Fmoc protecting group. (b) For the coupling reaction, DCC and 6-Cl-HOBt (0.20/0.21 mmol) were used to preactivate Fmoc-protected amino acids (0.21 mmol) in DMF at 25°C. (c) A cleavage cocktail consisting of TFA/H_2_O/TIPS/EDT (93/2/2.5/2.5 v/v/v) was applied for side-chain deprotection and peptide cleavage from the resin. (d) To generate the disulfide-linked cyclic structure, the linear peptide was subjected to oxidative folding in 10% DMSO-H_2_O solution for 24 h at 25°C (this step is specific to Lfcin DB synthesis). (e) The crude peptides were precipitated using cool diethyl ether, dried at 25°C, and analyzed via reverse-phase high-performance liquid chromatography (RP-HPLC) for purification and assessment (Lima et al., 2020).

### 2.3 Peptide purification and identification

The crude peptides were purified using solid-phase extraction (SPE) with a Supelclean LC-18 SPE column. The peptides were loaded onto the column, and the elution was carried out using a gradient of solvent B. The collected fractions were analyzed by RP-HPLC and mass spectrometry (MS). For RP-HPLC analysis, 20 μL of crude peptide stock solution (1 mg/mL) was injected onto a C18 column (Kromasil, 5 μm, 4.6 × 150 mm) using an Agilent 1200 liquid chromatograph (Agilent Technologies, NE, USA). A linear gradient from 20% to 50% solvent B (0.1% TFA in ACN) in solvent A (0.1% TFA in water) was applied over 25 min. Peptides were detected at 220 nm with a flow rate of 1.0 mL/min at 25°C. MALDI-TOF MS analysis was performed on an Ultraflex III TOF/TOF mass spectrometer (Bruker Daltonics, Bremen, Germany) in reflectron mode, using an MTP384 polished steel target (Bruker Daltonics) and 2,5-dihydroxybenzoic acid or sinapinic acid as the matrix. Data were acquired with 500 laser shots at 25%–30% power.

### 2.4 Circular dichroism spectroscopy

CD spectra of the peptides were collected using a CD spectropolarimeter (Applied Photophysics Ltd, UK). Each purified synthetic peptide was dissolved in H_2_O, PBS (5.8 mmol/L phosphate, 58.3 mmol/L NaCl, pH 7.0), PBS containing 0.56 mmol/L sodium dodecyl sulfate (SDS), or PBS containing 8.33 mmol/L SDS, to a final concentration of 0.16 mg/mL. The CD spectra were recorded at 25°C using a 0.5 mm pathlength quartz cuvette. Far-UV spectra were collected between 180 nm and 260 nm, with a step size of 1.0 nm. The bandwidth was set to 1.0 nm, and the response time was 0.5 s. Three successive spectra were accumulated and averaged for each sample. A blank spectrum of the buffer alone was measured and subtracted from each peptide spectrum to produce the final result. Mean residue ellipticity (MRE) [θ] was calculated using the following formula:


$$MRE\;=\;\left(\theta \;\times \;0.1\;\times \;MRW\right)/cl$$

where *θ* is the measured ellipticity in millidegrees (MDEG), *c* is the peptide concentration in mg/mL, *l* is the cuvette pathlength in cm, and MRW represents the mean residue weight of the peptide. MRW is defined as:


$$MRW\;=\;MW/\left(n\;–\;1\right)$$

where MW represents the molecular weight of the peptide, and *n* is the number of amino acid residues in the peptide.

### 2.5 Quantitative secondary structure analyses

All acquired CD spectral data files for the same peptide were imported into a single data interface within the CD analysis software (Chirascan), followed by pressing the “D” key to activate the processing module; the background signal was normalized by averaging two replicate CD spectra of ultrapure water (select both files > click “Average” to generate “Average:0”), while triplicate sample measurements were similarly averaged (select three sample files > “Average” to create “Average:1”); the background-corrected sample spectrum was obtained by selecting “Average:0” > clicking “Set Baseline,” then selecting “Average:1” > clicking “Subtract Baseline” to produce “Subtracted:0,” followed by deselection via “Unset Baseline”; finally, the “Subtracted:0” file was smoothed by selecting “Smooth,” applying a window size of 5 in the smoothing interface, confirming with “OK” to generate “Smooth(s):0” (smoothed spectrum) and “Smooth(r):0” (residual noise), retaining only the smoothed spectrum via “Remove Others” before final data export.

Secondary structure analysis of the CD data was performed using the CDNN software (Applied Photophysics Ltd.) by first opening the software and selecting “Options” in the menu bar, followed by choosing “Preferences” from the dropdown menu and selecting “Net using 33 basespectra (complex CD spectra)”; subsequently, the CD spectral data file was loaded by clicking “File” > “Open,” after which “Milli-Degrees” was selected under “CD Signal Type,” and essential parameters including “Molec. mass (Da),” “Cocentr. (mg/mL),” “No. amino acids,” and “Pathlength (cm)” were input under the “Essential Parameter” options; finally, clicking “OK” confirmed the settings, and the deconvolution was executed by clicking “Deconvolute” on the main interface to generate quantitative secondary structure data.

### 2.6 Tertiary structure prediction via AlphaFold3

The three-dimensional structures of Lfcin and its mutant type Lfcin C36G were predicted using AlphaFold3 (v3.0.0, DeepMind), a deep learning framework that synergizes evolutionary covariation patterns, physico-chemical constraints, and geometric deep learning to model protein folding (Josh et al., 2024). Monomeric peptide sequences in FASTA format were submitted to the AlphaFold3 Server (https://golgi.sandbox.google.com/) with the following parameterization: molecular entity type set to “protein,” oligomerization state defined as a single chain (copy_number = 1), and post-translational modifications explicitly disabled. To explore conformational diversity, eight independent models per target were generated by distinct seed values. Model selection prioritized structural confidence metrics: the highest-ranking model was chosen based on the composite evaluation of per-residue predicted Local Distance Difference Test (pLDDT) scores (threshold >80 for core regions) and interface Prediction-TM (ipTM) scores (>0.7 for inter-domain interactions), with priority given to architectures minimizing steric clashes and topological violations.

To establish experimental relevance, the predicted conformational ensembles were cross-validated against two experimentally resolved Lfcin structures: (1) the solution-state NMR structure (PDB: 1LFC), representing unconstrained peptide dynamics in aqueous buffer; and (2) the intramolecularly constrained Lfcin conformation structurally resolved within full-length lactoferrin by X-ray crystallography (PDB: 1BLF).

### 2.7 Susceptibility testing

To assess the role of the disulfide bond in the antibacterial activity of Lfcin, four bacterial strains, *E. coli* ATCC 25922, *K. pneumoniae* ATCC 4352, *P. aeruginosa* ATCC 27853, and *S. gallinarum* isolate were selected for further experimentation. Bacterial susceptibility was evaluated using agar disk diffusion (ADD) assays. Briefly, Pre-warmed MHA plates were seeded with 200 μL aliquots of bacterial inoculum (2 × 10^6^ CFU/mL), and standard antibiotics discs were placed onto the agar surface. The ADD assays were conducted using a single concentration of each peptide (200 μg/disc), followed by incubation at 37 °C for –24 h. Ciprofloxacin (1.25 μg/mL) was used as a positive control for all tested strains, while sterile water was used as a negative growth control. All susceptibility tests included three independent biological replicates per trial, with each biological replicate comprising three technical replicates to ensure reproducibility.

### 2.8 Antibacterial activity assays

Due to the absence of published minimum inhibitory concentration (MIC) breakpoints for AMP in the CLSI criteria or available literature, the susceptibility assays for the isolated strains were conducted using broth dilution methods that were previously described (Wiegand et al., 2008). Specifically, the broth microdilution assay was employed to determine the MIC and minimum bactericidal concentration (MBC). Briefly, each bacterial strain was inoculated in MHB and incubated at 200 rpm for 12 to 15 h at 37 °C until an optical density of 0.15 to 0.30 at 620 nm was reached. A 75 μL aliquot of peptide solution (4 mg/mL) was added to the first column of a 96-well polypropylene microtiter plate (Costar 3790, Corning Inc., USA). Each row was then serially diluted two-fold using 2×AABSA solution (0.2 μL/mL acetic acid, 4 mg/mL BSA) from the first to the tenth column. The peptide solution was mixed with an equal volume of bacterial inoculum (1 × 10^6^ CFU/mL) in each well. The final volume of each well is 150 μL. This resulted in final peptide concentrations of 1000, 500, 250, 125, 62.5, 31.25, 15.63, 7.81, 3.91, and 1.95 μg/mL, while the bacterial concentration was adjusted to 5 × 10^5^ CFU/mL, consistent with previous findings (Wiegand et al., 2008). The inoculated plates were incubated with shaking at 200 rpm for 24 h at 37 °C, and absorbance was measured at 620 nm using an Asys Expert Plus ELISA reader.

MIC_50_, MIC_90_, and MBC were determined as follows: A 100 μL aliquot from each well was serially ten-fold diluted. Dilutions were then spread on MHA plates and incubated at 37°C for 24 h. Plates yielding less 300 colonies were selected to calculate the original CFU/mL of cultured bacteria, which was used to derive MIC_50_, MIC_90_, and MBC values. MIC_50_ was defined as the minimum concentration of the antimicrobial peptide required to inhibit growth of ≥ 50% of bacterial colonies compared to peptide-free control plates. MIC_90_ was defined as the minimum concentration of the antimicrobial peptide required to inhibit growth of ≥ 90% of bacterial colonies compared to peptide-free control plates. MBC was defined as the lowest peptide concentration that results in a ≥ 99.9% reduction in bacterial counts compared to untreated controls (Wiegand et al., 2008; Kim, 2010). Three independent antibacterial activity assays were conducted at one-week intervals using freshly cultured bacterial suspensions. Each experiment included triplicate technical measurements to ensure intra-assay precision.

### 2.9 Statistical Analysis

The experimental design incorporated three independent biological replicates (distinct cultures) with three technical replicates per condition. Data are expressed as mean ± standard deviation. Between-group comparisons were analyzed using a Student’s t-test, with statistical significance defined as p < 0.05. All analyses, including data visualization, were conducted in R software (version 4.4.2, R Core Team, 2024).

## 3 Results

### 3.1 Peptide syntheses and purifications

Bovine Lfcin, Lfcin DB, and Lfcin C36G peptides were successfully synthesized using solid phase peptide synthesis (SPPS). The purification process, conducted through solid-phase extraction chromatography (SPEC), ensured high-quality peptides, each achieving over 95% purity as confirmed by RP-HPLC. The structural schematics of these peptides are displayed in **Figure 1A**, and their molecular weights, verified through matrix assisted laser desorption/ionization-time of flight-mass spectrometry (MALDI-TOF-MS), were consistent with the expected values. The molecular structures are illustrated in **Figure 1B**, while **Table 1** provides a detailed list of all synthetic peptides used in this study.

**Table 1:** Synthetic peptides tested for antibacterial activity.

Peptides	Amino acid sequences (N terminal to C terminal)	Length (AA)	Molecular weight	Purity (%)
Lfcin	FKCRRWQWRMKKLGAPSITCVRRAF	25	3125.82	>95
Lfcin DB	FKCRRWQWRMKKLGAPSITCVRRAF (with a disulfide bond)^a^	25	3123.82	>95
Lfcin C36G	FKCRRWQWRMKKLGAPSITGVRRAF^b^	25	3079.73	>95

^a^The cysteines forming a disulfide bond are shadowed. ^b^The glycine mutation from the cysteine is boxed.

### 3.2 Circular dichroism spectra

In far-UV CD spectroscopy (180–260 nm), protein analysis typically requires concentrations between 0.1-0.5 mg/mL to achieve measurable signal-to-noise ratios. Through preliminary CD optimization trials with Lfcin and its derivatives, we empirically determined that 0.16 mg/mL provided the optimal compromise between spectral signal quality and baseline stability for these specific peptides under our experimental conditions.

**Figure 2:**
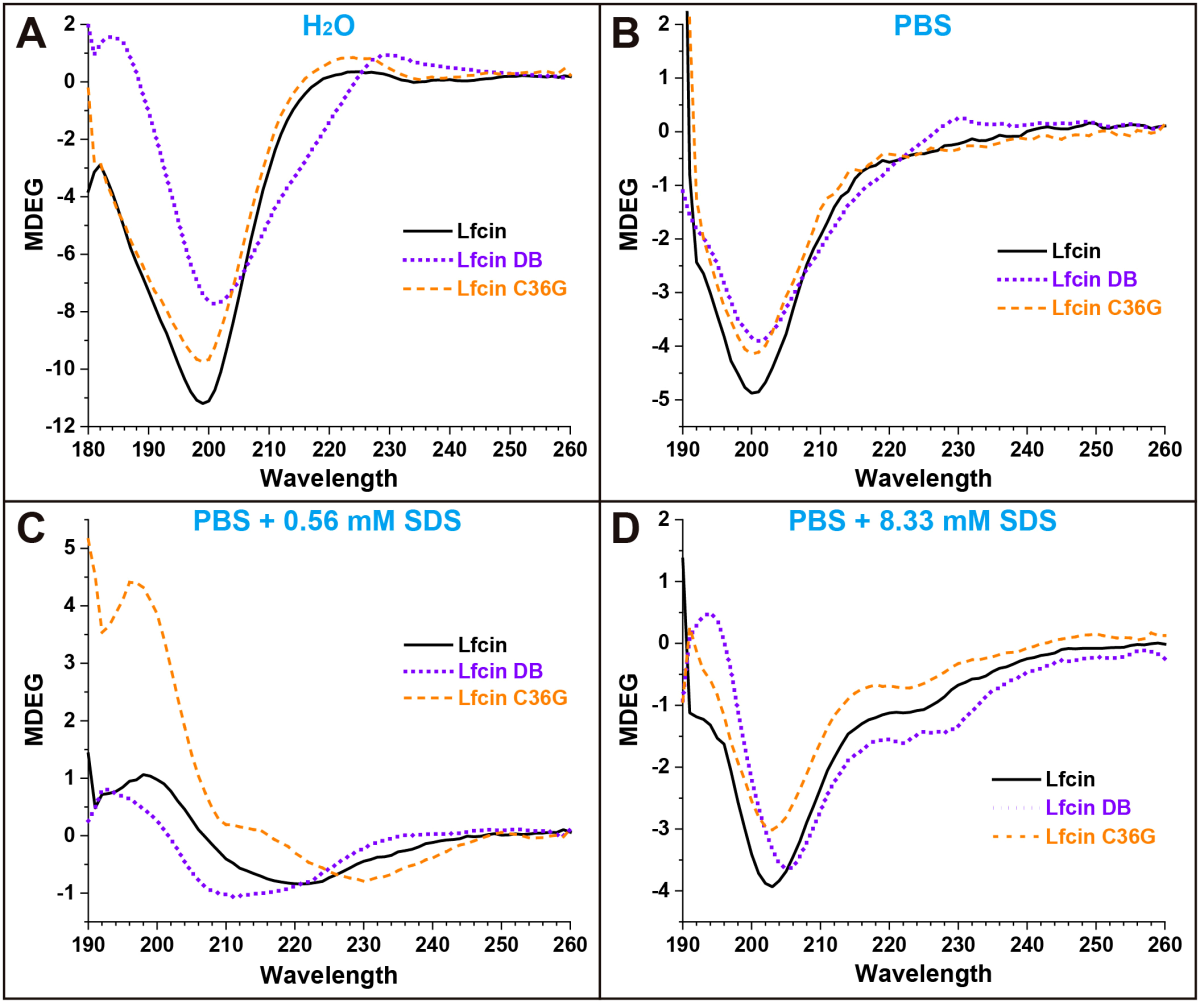
CD spectra of Lfcin and its derivatives in various solutions. The peptides (0.16 mg/mL) were tested in four solution conditions: **(A)** deionized H_2_O, **(B)** PBS, **(C)** PBS containing 0.56 mM SDS, **(D)** PBS containing 8.33 mM SDS. Different colored lines represent CD spectra, with the x-axis showing the measured wavelength and the y-axis indicating ellipticity in millidegrees.

**Figure 2** presents the CD spectra curves for the three synthesized peptides under various conditions. In H_2_O, Lfcin and Lfcin C36G displayed similar spectral profiles, with ellipticity (measured in MDEG) reaching a minimum around 199 nm, as shown in **Figure 2A**. In contrast, Lfcin DB exhibited a higher ellipticity, with its lowest point occurring at approximately 201 nm. In PBS, all three peptides demonstrated comparable waveform patterns, with minima around 200 nm, although Lfcin had the lowest ellipticity among the group (**Figure 2B**). When measured in PBS containing 0.56 mM SDS, the peptides exhibited maxima between 192 and 196 nm. The MDEG profiles of Lfcin and Lfcin DB showed similar patterns, with Lfcin DB displaying a slight leftward shift (**Figure 2C**). In the presence of a higher SDS concentration (8.33 mM), all three peptides showed similar MDEG curves with minima ranging from 202 nm to 206 nm, indicating consistent structural behavior across different peptides (**Figure 2D**).

### 3.3 Secondary structure ratios

**Figure 3:**
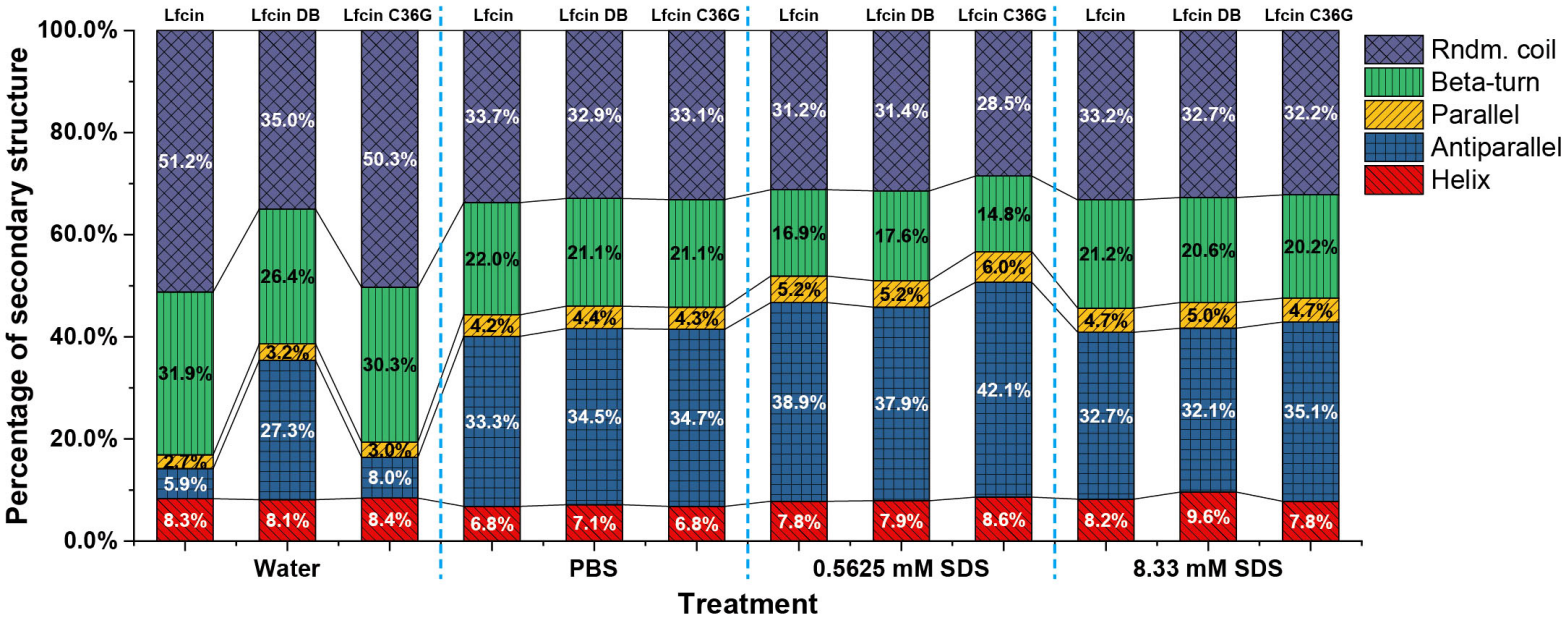
Percentages of secondary structures for Lfcin and its derivatives in distinct solutions. The peptides were tested in four solution conditions: deionized H_2_O; PBS, PBS containing 0.56 mM SDS, PBS containing 8.33 mM SDS. Various structural types are represented in different colors: Rndm. Coil (random coil), Beta-turn, Parallel (parallel β-sheet), Antiparallel (antiparallel β-sheet), and Helix (α-helix).

**Figure 3** illustrates the secondary structure ratios of the three synthesized peptides. In H_2_O, Lfcin and Lfcin C36G exhibited a clear preference for random coil and β-turns structures, while Lfcin DB predominantly adopted an antiparallel β-sheet conformation. Notably, the random coil content in Lfcin and Lfcin C36G exceeded 50%, indicating that these two peptides primarily existed in irregular structural forms (**Figure 3**). When the peptides were analyzed in PBS, the secondary structure ratios showed a high degree of similarity across all three peptides, suggesting they shared comparable overall protein conformations (**Figure 3**). In PBS containing 0.56 mM SDS, Lfcin and Lfcin DB exhibited similar distributions of secondary structure elements. However, Lfcin C36G showed a higher proportion of antiparallel β-sheets and lower percentages of parallel β-sheets and random coil structures compared to the other two peptides (**Figure 3**). At a higher SDS concentration (8.33 mM), all three peptides demonstrated nearly identical secondary structure ratios. Under these conditions, each peptide displayed an increased proportion of α-helix structures, with the remaining secondary structures, such as β-sheets and random coils, distributed more evenly, in contrast to their structural preferences in PBS alone (**Figure 3**).

### 3.4 Tertiary structure predictions

**Figure 4:**
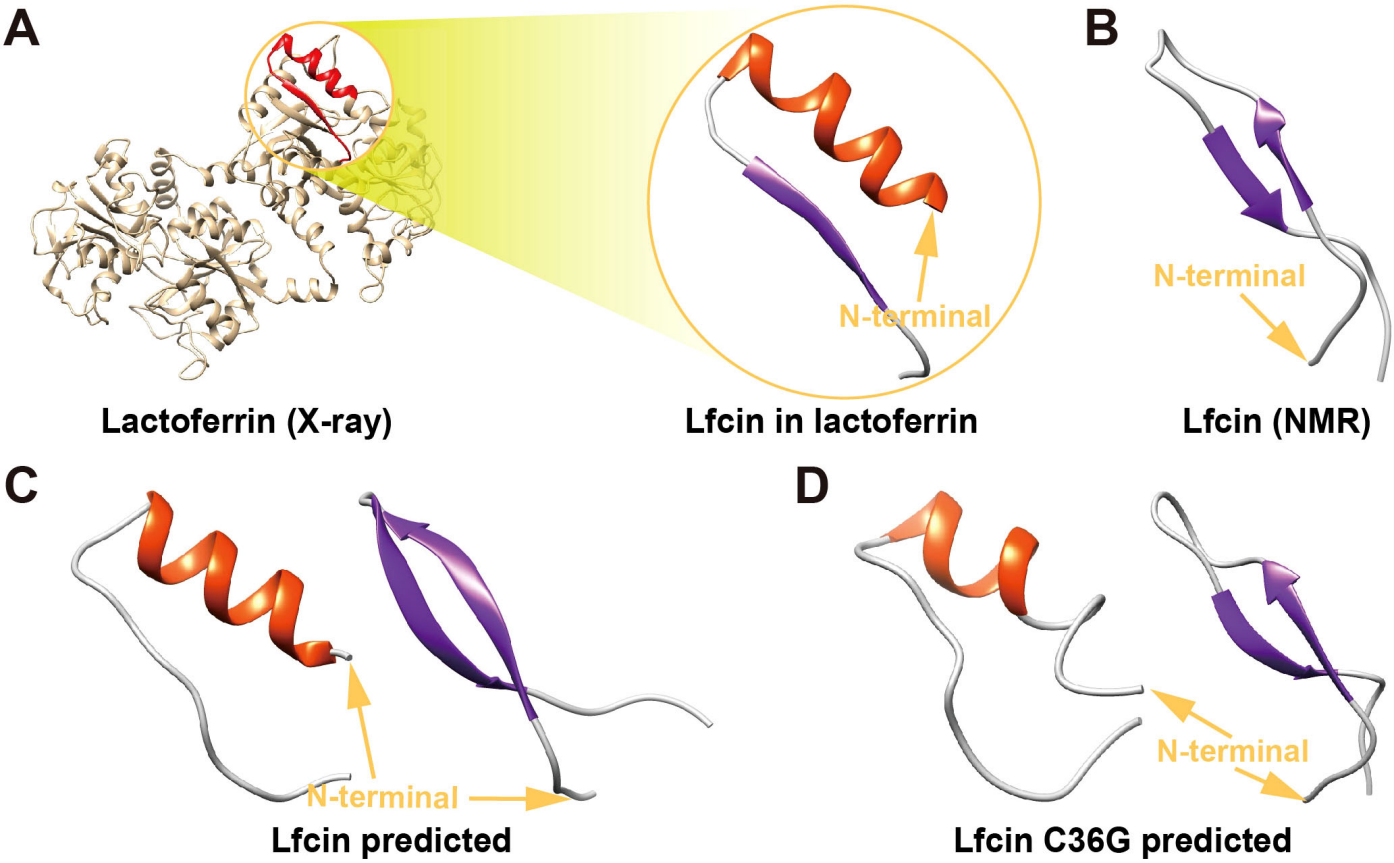
Tertiary structures of Lfcin and its C36G variant. **(A)** Native Lfcin structure, derived from X-ray crystallography analysis of full-lenth lactoferrin (PDB ID: 1BLF). **(B)** Solution-state Lfcin structure determined by nuclear magnetic resonance spectroscopy (PDB ID: 1LFC). **(C)** Predicted teriary structures of wild-type Lfcin generated by AlphaFold3. **(D)** Predicted tertiary structure of Lfcin C36G modeled by AlphaFold3. Structural orientations are indicated by golden N-terminal arrows. The secondary structural elements are color-coded: α-helices in red and β-sheets in blue.

AlphaFold3 predictions resolved two thermodynamically distinct conformational ensembles for both Lfcin and its C36G variant: α-helix-rich conformers characterized by a well-defined α-helix locating the N-terminal half with a disordered C-terminal tail, and β-sheet-dominant conformers comprising antiparallel β-strands at both termini interconnected by a central flexible coil (**Figures 4C, D**).

Notably, the α-helix-rich models of wild-type Lfcin displayed 57% higher helical content compared to Lfcin C36G (11 vs. 7 residues), while β-sheet-dominant conformers of Lfcin contained 50% more β-strand residues than the mutant (12 vs. 8 residues). These results suggest that the mutant’s reduced propensity for ordered structure formation.

The β-sheet-predicted architectures showed high congruence with the NMR-derived Lfcin structure (PDB: 1LFC, RMSD = 1.3 Å), particularly in β-strand topology and coil orientation (**Figure 4B**). Conversely, α-helix-rich models aligned with the X-ray crystallography structure of lactoferrin-embedded Lfcin (PDB: 1BLF, RMSD = 2.8 Å) in helical registry, though the latter adopts a C-terminal β-sheet motif absent in our isolated peptide predictions (**Figure 4A**). This discrepancy indicates that the embedded Lfcin β-sheet conformation is stabilized by intermolecular interactions within full-length lactoferrin.

### 3.5 Inhibition halo analyses

**Figure 5:**
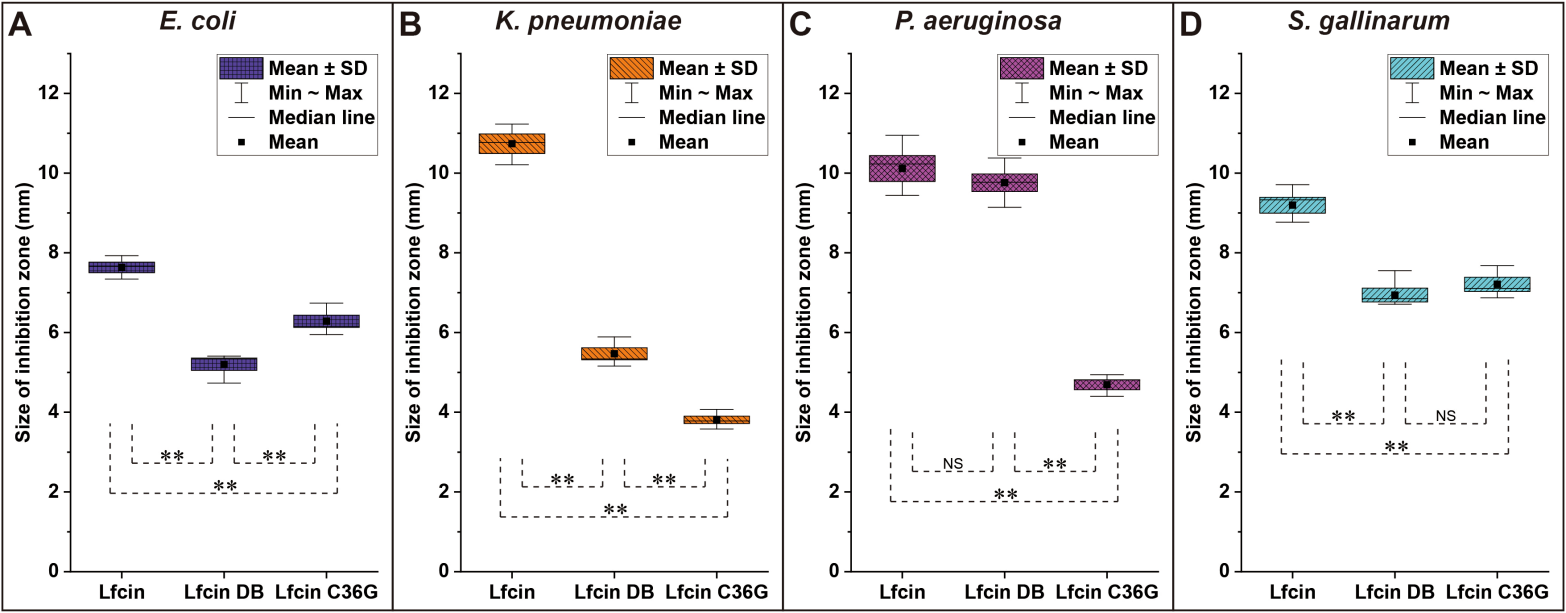
Inhibition zones of bLfcin and its derivatives against tested bacteria in susceptibility assays. Disc diffusion tests were conducted for *E. coli* **(A)**, *K. pneumoniae* **(B)**, *P. aeruginosa* **(C)**, and *S. gallinarum* **(D)**. Sterile discs were placed on MH agar plates inoculated with the strains, and 20 μL of each peptide (20 mg/mL) was added to the discs. Plates were incubated at 37°C for 24 h. The size of the inhibition zone was calculated as (diameter of halo – diameter of disc) × 1/2. Each assay was performed in triplicate, with statistically significant differences noted (**P < 0.01).

In the susceptibility assays, Lfcin demonstrated the largest inhibition halo sizes against *E. coli* compared to the other two peptides, with Lfcin C36G showing larger halos than Lfcin DB. A similar pattern was observed against *S. gallinarum*, although in this case, Lfcin DB produced slightly larger halos than Lfcin C36G. Against *K. pneumoniae*, Lfcin once again exhibited the most significant inhibition, with Lfcin DB producing larger halos than Lfcin C36G. A comparable trend was noted for *P. aeruginosa*, analogously, where both Lfcin and Lfcin DB generated larger inhibition zones than Lfcin C36G, with Lfcin and Lfcin DB showing halos of similar size (**Figure 5**).

### 3.6 MICs and MBCs

The synthetic peptides demonstrated antibacterial activity against *E. coli*, *K. pneumoniae*, *P. aeruginosa*, and *S. gallinarum*, with MIC and MBC values ranging from 15.6 to 500.0 μg/mL (**Table 2**). Consistent with the inhibition halo experiments, Lfcin exhibited superior antibacterial efficacy against all tested bacterial strains compared to its derivatives, Lfcin DB and Lfcin C36G (**Table 2**).

**Table 2:** Antibacterial activity of the designed synthetic peptides against the four Gram-negative bacteria.

Strain	Antibacterial index	Lfcin	Lfcin DB	Lfcin C36G
*Escherichia coli* ATCC 25922	MIC_50_^a^	62.5 (20.0)^b^	125.0 (40.0)	125.0 (40.6)
	MIC_90_^a^	62.5 (20.0)	250.0 (80.0)	125.0 (40.6)
	MBC	62.5 (20.0)	250.0 (80.0)	125.0 (40.6)
*Klebsiella pneumoniae* ATCC 4352	MIC_50_	15.6 (5.0)	125.0 (40.0)	250.0 (81.2)
	MIC_90_	15.6 (5.0)	250.0 (80.0)	500.0 (162.4)
	MBC	15.6 (5.0)	250.0 (80.0)	500.0 (162.4)
*Pseudomonas aeruginosa* ATCC 27853	MIC_50_	15.6 (5.0)	15.6 (5.0)	250.0 (81.2)
	MIC_90_	31.3 (10.0)	31.3 (10.0)	250.0 (81.2)
	MBC	31.3 (10.0)	31.3 (10.0)	250.0 (81.2)
*Salmonella gallinarum* isolates	MIC_50_	31.3 (10.0)	62.5 (20.0)	62.5 (20.3)
	MIC_90_	31.3 (10.0)	125.0 (40.0)	125.0 (40.6)
	MBC	31.3 (10.0)	125.0 (40.0)	125.0 (40.6)

^a^The MIC_50_ and MIC_90_ represent the concentrations required to inhibit 50% and 90% of the strains, respectively; ^b^MIC_50_, MIC_90_, and MBC in μg/mL (μM).

For *E. coli*, Lfcin showed the strongest antibacterial activity, with MIC_50_, MIC_90_, and MBC values all at 62.5 μg/mL. Both Lficn DB and Lfcin C36G had a MIC_50_ of 125.0 μg/mL, but Lfcin DB exhibited higher MIC_90_ and MBC values (250.0 μg/mL) than Lfcin C36G, whose values were 125.0 μg/mL.

Against *K. pneumoniae*, Lfcin again demonstrated the most potent antibacterial effect, with MIC_50_, MIC_90_, and MBC all measured at 15.6 μg/mL. Lfcin C36G displayed a higher MIC_50_ (250 μg/mL) compared to Lfcin DB (125 μg/mL). Moreover, Lfcin C36G had the same MIC_90_ and MBC (500 μg/mL), both of which were higher than those of Lfcin DB (250 μg/mL).

In the case of *P. aeruginosa*, Lfcin and Lfcin DB exhibited comparable antibacterial potency, with both peptides having a MIC_50_ of 15.6 μg/mL, which was markedly lower than Lfcin DB’s MIC_50_ of 250 μg/mL. Additionally, Lfcin and Lfcin DB shared identical MIC_90_ and MBC values of 31.3 μg/mL, significantly lower than those of Lfcin C36G (250 μg/mL).

Similarly, for *S. gallinarum*, Lfcin exhibited the most robust antibacterial activity, with MIC_50_, MIC_90_, and MBC values consistently at 31.3 μg/mL. Both Lfcin DB and Lfcin C36G showed identical MIC_50_ values of 62.5 μg/mL, as well as matching MIC_90_ and MBC values of 125 μg/mL.

## 4 Discussion

### 4.1 Structural insights of Lfcin derived from circular dichroism spectra

The CD spectra of Lfcin and its derivatives provide valuable insights into their secondary structures across different solvent environments (**Figure 2**). In this study, the CD spectra of Lfcin closely resembled those in a previous report (Huertas Méndez et al., 2017), despite being recorded under varying conditions. In aqueous solution, Lfcin and its derivatives predominantly exhibited a random coil conformation (**Figure 3**). However, in phosphoric acid solutions, there was a notable decrease in random coil ratios alongside an increase in well-structured conformations (**Figure 3**). This suggests that the saline ions may facilitate the proper folding of these polypeptides, potentially mimicking their behavior under physiological ionic conditions. Among the tested solutions, β-turns were most pronounced in water (**Figure 3**), aligning with previous findings that showed Lfcin17–30 exhibited more defined β-turns in lower ionic strength buffers compared to higher ionic strength buffers (Bolscher et al., 2009). Furthermore, a greater proportion of twisted β-sheet amphipathic structures was observed for Lfcin and its derivatives in water (**Figure 3**), consistent with earlier studies (Hwang et al., 1998). In the presence of SDS, Lfcin and its derivatives transitioned towards α-helix or β-sheet conformations (**Figure 3**), demonstrating their adaptability to hydrophobic microenvironments (Yeaman and Yount, 2003). This structural flexibility is vital for effective membrane insertion and disruption, as it allows AMPs to reorient themselves for optimal interaction with microbial membranes (Sani and Separovic, 2016; Chen et al., 2023).

### 4.2 Secondary structural transformations of Lfcin

The critical micelle concentration (CMC) is the threshold concentration at which surfactants, such as SDS, begin to aggregate and form micelles in solution (Zhong et al., 2015). SDS achieves this at a CMC of approximately 8 mM in aqueous environments, creating membrane-mimetic structures. In these micelles, hydrophobic groups cluster to form an inner core, while hydrophilic portions, including negatively charged sulfonic acid groups, compose the outer shell, resembling the negatively charged surfaces of bacterial membranes.

Lfcin interacts with bacterial plasma membranes primarily through electrostatic attractions between its positively charged residues and the negatively charged components of the bacterial surface (Huertas Méndez et al., 2017). In our study, we observed that the three peptides displayed similar MDEG curve profiles in both PBS and PBS containing 8.33 mM SDS (**Figure 3**), indicating that the formation of disulfide bonds did not significantly alter their secondary structures in either environment. Interestingly, there was a slight increase in the proportion of helical structures for all peptides in the presence of 8.33 mM SDS (**Figure 3**), suggesting that SDS micelles facilitate the adoption of helical conformations over other secondary structures.

When SDS micelles are diluted below the CMC, they disassemble into free surfactant molecules, impacting the peptide structures through interactions with monomeric SDS (Lu et al., 2018). At a much lower concentration of 0.56 mM SDS, we noted that the peptides exhibited an increased ratio of antiparallel structures and a reduction in β-turns (**Figure 3**). Notably, Lfcin and Lfcin DB showed similar secondary structure ratios, while Lfcin C36G displayed a higher proportion of antiparallel configurations and fewer β-turns (**Figure 3**), indicating a propensity for disulfide bond formation in hydrophobic conditions.

Among the four solutions tested, Lfcin DB exhibited the least structural variation (**Figure 3**), suggesting that its intra-chain disulfide bond restricts its conformation within a limited spatial range. This confinement results in relatively stable secondary structures across varying ionic strengths and hydrophobic environments. In particular, Lfcin DB demonstrated more organized structures in water compared to the other peptides, highlighting the influence of disulfide bonding on structural integrity. The secondary structure ratios (e.g., α-helix and β-sheet content) Lfcin B and its derivatives closely align with those observed in a prior study (Pei et al., 2021), underscoring the robustness of the analytical methods employed for peptide structural characterization.

### 4.3 Predicted tertiary structure comparisons

The striking congruence between β-sheet-predicted conformers and the NMR solution structure (PDB: 1LFC) (Hwang et al., 1998) suggests these architectures represent physiologically relevant states in aqueous environments (**Figure 4B**). Conversely, the moderate alignment of α-helical models with the X-ray lactoferrin-embedded structure (PDB: 1BLF) (Moore et al., 1997) reveals context-dependent folding: the chimeric α/β topology observed in 1BLF likely requires intramolecular constraints, which stabilizes non-native β-strands through hydrogen-bond networking absent in isolated peptides (**Figure 4A**). This dichotomy highlights Lfcin’s conformational adaptability—adopting helical states as free peptides versus β-enriched folds when structurally confined—a property potentially exploited for multifunctional interactions with diverse molecular targets.

The structural plasticity of Lfcin and its C36G mutant, as resolved by AlphaFold3, underscores the critical role of residue-level interactions in dictating conformational equilibria (**Figures 4C, D**). The marked reduction in α-helical (57%) and β-strand (50%) content in C36G compared to wild-type Lfcin directly implicates Cys36 as a crucial stabilizer of ordered secondary structures. This residue likely participates in a conserved hydrophobic cluster that reinforces helical packing in the N-terminal domain—a feature disrupted by the C36G substitution, leading to enhanced conformational entropy in the mutant. Such structural destabilization may decrease antimicrobial potency, as disordered conformers could reduce stability, membrane permeability, and binding affinity (Muttenthaler et al., 2021).

### 4.4 Alterations in antibacterial activity of Lfcin

The normalization of bacterial cell numbers in susceptibility testing is essential for achieving accurate and reproducible results (Dadura et al., 2017). Reports indicate that Lfcin exhibits varying antibacterial activity depending on the testing method used. For instance, against *E. coli* IID861, Lfcin displayed a MIC of 50 μg/mL in MHB, contrasting with an MIC of 6 μg/mL in peptone-based broth (Leon-Calvijo et al., 2015). This discrepancy may be linked to the inoculum concentration utilized for determining the MIC/MBC (Oo et al., 2010). Specifically, a higher inoculum concentration can lead to an increased MIC if the bacterium produces enzymes that degrade the antimicrobial agent, while a lower concentration than recommended may result in artificially reduced MICs (Wiegand et al., 2008). To address these variations caused by different antibacterial methods, the protocols in this study were standardized following the methodology established by previous research (Wiegand et al., 2008).

The results of this study indicate that the MIC of Lfcin (62.5 μg/mL) aligns with previous reports on its antibacterial activity against *E. coli* ATCC 25922 (**Table 2**), which ranged from 30 to 100 μg/mL (Leon-Calvijo et al., 2015; Bruni et al., 2016; Huertas Méndez et al., 2017). The susceptibility profiles of *K. pneumoniae* strains to Lfcin and its analogs revealed notable strain-specific variability. Specifically, *K. pneumoniae* ATCC 4352 demonstrated marked sensitivity to Lfcin, with MIC of 15.6 μg/mL in our study (**Table 2**), indicating potent bactericidal activity against this strain. In contrast, *K. pneumoniae* JCM-1662T exhibited reduced susceptibility, requiring a significantly higher MIC for Lfcin as previously reported (Bellamy et al., 1992a). These discrepancies likely arise from intrinsic differences between the bacterial strains employed in the assays. The ATCC 4352 strain may possess distinct membrane permeability or efflux pump activity compared to JCM-1662T. Genetic divergence, such as variations in lipopolysaccharide (LPS) structure, capsular serotypes, or differential expression of resistance genes, could further modulate interactions with cationic antimicrobial peptides like Lfcin.

The observed MIC of Lfcin against *P. aeruginosa* ATCC 27853 in this study was 62.5 μg/mL (**Table 2**), which contrasts sharply with the previously reported MIC of 5 μg/mL for a *P. aeruginosa* strain isolated in another laboratory (Nawal Abd et al., 2021). This discrepancy is likely attributable to inherent genomic and phenotypic variations between the *P. aeruginosa* strains employed in these experiments. While ATCC 27853 is a standardized reference strain widely used for antimicrobial susceptibility testing and quality control, the strain described by Nawal Abd et al. may represent a clinical isolate with distinct resistance profiles or adaptive mechanisms. Future investigations should prioritize comparative genomic analyses of *P. aeruginosa* strains to identify molecular mechanisms underlying Lfcin resistance, particularly in hypervirulent or multidrug-resistant clinical isolates.

This study demonstrates the strong antibacterial activity of native Lfcin and its derivatives against multidrug-resistant (MDR) pathogens. Previous research underscores the significance of Lfcin’s amphipathic structure, characterized by the arrangement of hydrophobic and cationic residues, which facilitates effective membrane insertion and disruption through electrostatic interactions with negatively charged bacterial membranes (Haney et al., 2009; Lei et al., 2021; Semeraro et al., 2022). This structural feature allows Lfcin to interact effectively with bacterial membranes, ultimately leading to membrane disruption and cell death (Sinha et al., 2013). Such mechanisms align with the “carpet model,” wherein peptides align parallel to the membrane surface, compromising membrane integrity upon reaching a critical concentration (Nguyen et al., 2011; Islam et al., 2023).

Lfcin exhibited significantly greater antibacterial activity against all tested pathogens compared to its derivatives, as indicated by larger inhibition zones and lower MICs, and MBCs (**Figure 5**, **Table 2**). Notably, Lfcin demonstrated structural similarities to Lfcin C36G in aqueous conditions and to Lfcin DB in hydrophobic environments (**Figure 3**). These findings suggest that Lfcin can switch between macrocyclic and linear conformations, enhancing its antibacterial efficacy. While neither conformation alone exhibited superior activity, the ability of Lfcin to transform is advantageous for maximizing its antibacterial function. It is hypothesized that Lfcin forms disulfide bonds in hydrophobic conditions, which may be disrupted when ionic and hydrophilic interactions are reduced. This implies that an oxidizing environment could exist near the hydrophobic regions of bacterial membranes, facilitating disulfide bond formation. In contrast, Lfcin C36G was unable to adopt a macrocyclic conformation similar to that of Lfcin and Lfcin DB under ionic and hydrophobic conditions.

The addition or removal of cysteine residues significantly influences the structural flexibility and stability of peptides, particularly through the formation of disulfide bonds (Li et al., 2012). Disulfide bonds can enhance peptide stability against proteolytic degradation (Haney and Hancock, 2013; Morris, 2019). Our findings indicate that Lfcin DB, which features a disulfide bond between Cys19 and Cys36, shows enhanced conformational stability compared to the native peptide, as evidenced by reduced secondary structure variation across all tested solution conditions (**Figure 3**). While disulfide bonds are known to enhance stability, membrane permeability, and binding affinity (Muttenthaler et al., 2021), Lfcin DB displayed weaker antibacterial activity than Lfcin. This reduced efficacy may be attributed to Lfcin DB’s more stable β-sheet conformation, which likely hinders its ability to disrupt membranes effectively, as a less flexible structure could impede penetration (Andersson et al., 2016). Consequently, the presence of the disulfide bond may limit the necessary structural flexibility for optimal antibacterial function (Haney and Hancock, 2013). These findings support the hypothesis that bovine Lfcin may undergo structural changes via intramolecular disulfide bond formation or cleavage, thereby modulating its antibacterial activity through altered interactions with bacterial membranes. Future research should focus on finding a balance between enhancing peptide stability and maintaining membrane-disrupting capability to improve the therapeutic potential of Lfcin derivatives. While rigid, disulfide-stabilized peptides prevent structural instability, they exhibit limited conformational flexibility required for dynamic interactions with microbial membranes. Conversely, flexible linear peptides achieve potent antibacterial activity through enhanced membrane penetration but suffer from reduced stability under physiological stressors. Through multiparameter optimization integrating structural rigidity and dynamic adaptability, we should systematically reconcile this tradeoff, guided by structure-activity relationship principles to design peptides that synergize therapeutic durability and potency.

### 4.5 Limitations

While the effects of membrane-mimetic environments on the molecular structures of Lfcin and its two derivatives were analyzed, these conditions cannot fully replicate the complexity of actual bacterial membranes, which have a far more intricate lipid composition. Additionally, current technologies do not allow for direct assessment of Lfcin and its derivatives on the bacterial surface using CD spectra, due to the diverse and complex lipid environments present in bacterial membranes. Moreover, the formation of disulfide bonds within Lfcin on the bacterial membrane surface remains unclear from the findings of this study. Despite these limitations, our research confirmed that Lfcin can undergo structural changes in response to variations in ionic strength and hydrophobicity, which are likely to enhance its antibacterial activity.

## 5 Conclusions

This study demonstrated that bovine Lfcin can alter its molecular conformation in response to varying ionic strengths and hydrophobic conditions. Both Lfcin and its derivatives showed significant antibacterial activity against *E. coli*, *K. pneumoniae*, *P. aeruginosa*, and *S. gallinarum*, with the native Lfcin peptide exhibiting the highest efficacy. The dynamic transition between linear and cyclic conformations enhances Lfcin’s potent antimicrobial properties. While the introduction of disulfide bonds can improve peptide stability, it reduces antibacterial effectiveness by limiting structural flexibility. These findings underscore Lfcin’s potential as a template for developing new antimicrobial therapies, emphasizing the need for further optimization to balance stability and efficacy in combating antibiotic-resistant infections. The present study highlights the potential of bovine Lfcin for further optimization through structure-based design strategies.

## Data Availability

The original contributions presented in the study are included in the article/**Supplementary Material**. Further inquiries can be directed to the corresponding author/s.
