# Supplementary material for: Bovine lactoferricin exerts antibacterial activity against four Gram-negative pathogenic bacteria by transforming its molecular structure
Source: Front Cell Infect Microbiol. 2025 May 16;15:1508895. doi: 10.3389/fcimb.2025.1508895 (PMC12122761; doi:10.3389/fcimb.2025.1508895)
Supplement: Supplementary file 1 [file DataSheet1.pdf]

Supplementary Material for HPLC and MS of Lfcin, Lfcin DB, and Lfcin C36G

Article Title

Bovine lactoferricin exerts antibacterial activity against four gram-negative pathogenic bacteria by transforming its molecular structure

Journal Name

Frontiers in Cellular and Infection Microbiology

Author names

Jie Pei, Lin Xiong, Xiaoyun Wu, Min Chu, Pengjia Bao, Qianyun Ge and Xian Guo

Affiliation

Key Laboratory of Yak Breeding in Gansu Province, Lanzhou Institute of Husbandry and Pharmaceutical Sciences, Chinese Academy of Agricultural Sciences, Lanzhou, Gansu, China; Key Laboratory of Animal Genetics and Breeding on Tibetan Plateau, Ministry of Agriculture and Rural Affairs, Lanzhou, Gansu, China

E-mail address of the corresponding author

guoxian@caas.cn

Lfcin HPLC report

Product Name : Lfcin FF-25-1
Instrument No.: 03019
Lot No. : P151123-LR488174
Column : 4.6*250mm, Kromasil C18 5um
Solvent A : 0.1% trifluoroacetic in 100% acetonitrile
Solvent B : 0.1% trifluoroacetic in 100% water
Gradient

	A	B
0.01min	22%	78%
25.0min	47%	53%
25.1min	100%	0%
30.0min	STOP	

Flow rate : 1.0ml/min
Wavelength : 220nm
Volume : 20ul

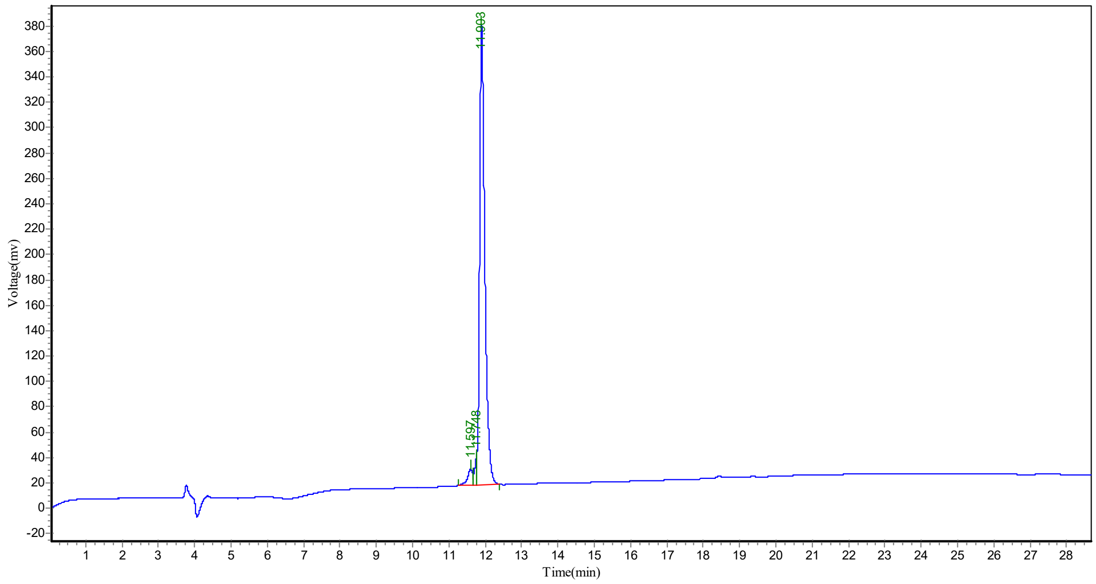

Rank	Time	Height	Area	Conc.
1	11.597	12452.989	126118.758	3.2887
2	11.748	20472.971	62723.043	1.6356
3	11.903	361771.313	3646018.500	95.0757
Total				100

MS Spectrum

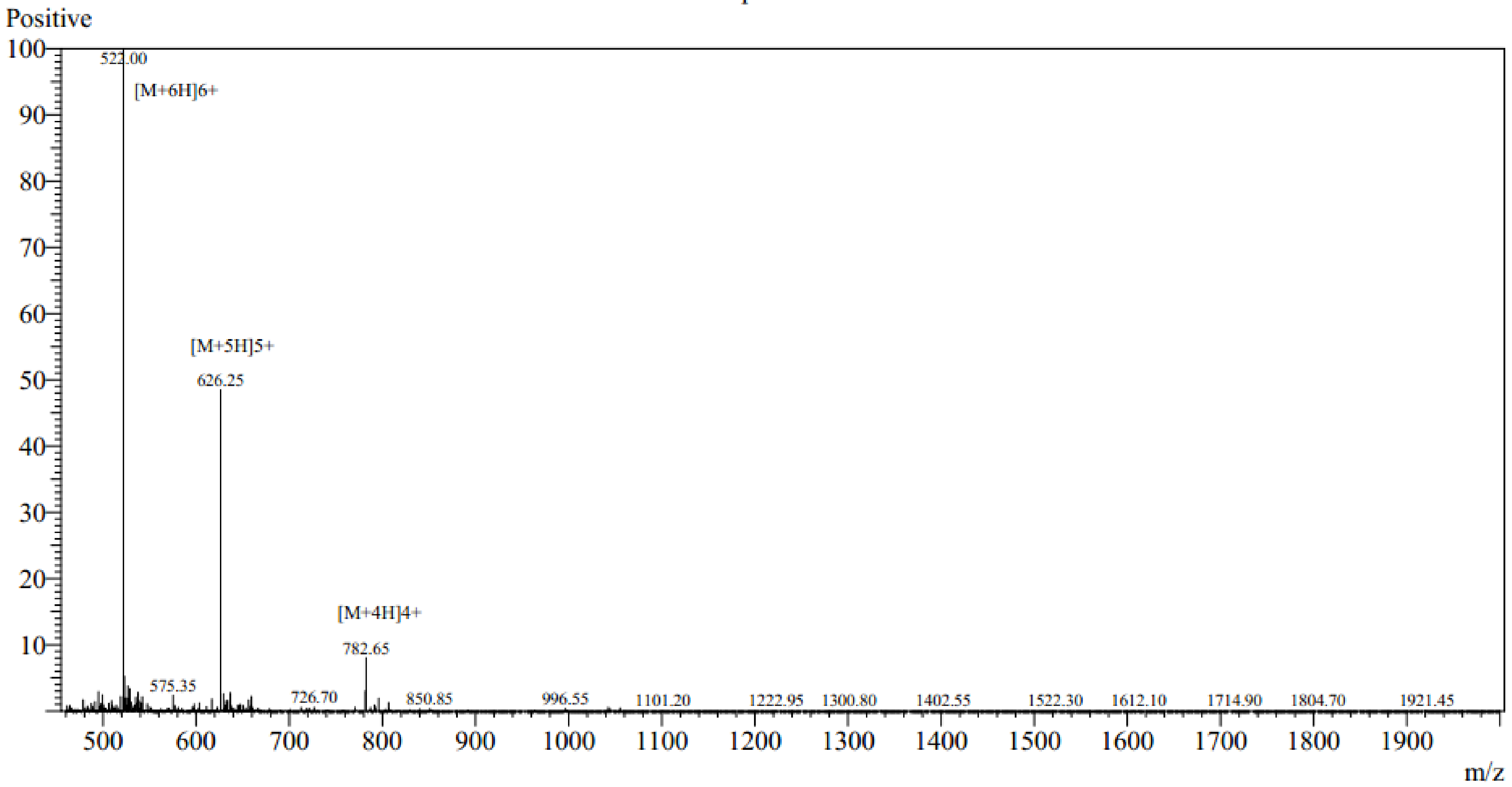

Acquired by	: Qiu	Probe	: ESI	Probe bias	: +4.5kv
Data Acquired	: 2018-12-16 11:55:24	Nebulizer Gas Floe	: 1.5L/min	Detector	: 2.0kv
Injection Volume	: 1	CDL	: -20.0v	T.Flow	: 0.2ml/min
Sample Name	: Lfcin FF-25-1	CDL Temp	: 250℃	B.conc	: 50%H2O/50%ACN
Mw	: 3125.84	Block Temp	: 200℃		
Lot No.	: P151123-LR488174				

Lfcin DB HPLC report

Product Name : Lfcin DB FF-25(0)
Instrument No.: 0306006
Lot No. : P151123-LR488159
Column : 4.6*250mm, Venusil XBP-C18(L) 5um
Solvent A : 0.1% trifluoroacetic in 100% acetonitrile
Solvent B : 0.1% trifluoroacetic in 100% water
Gradient

	A	B
0.01min	30%	70%
25.00min	55%	45%
25.10min	100%	0%
30.0min	STOP	

Flow rate : 1.0ml/min
Wavelength : 220nm
Volume : 10ul

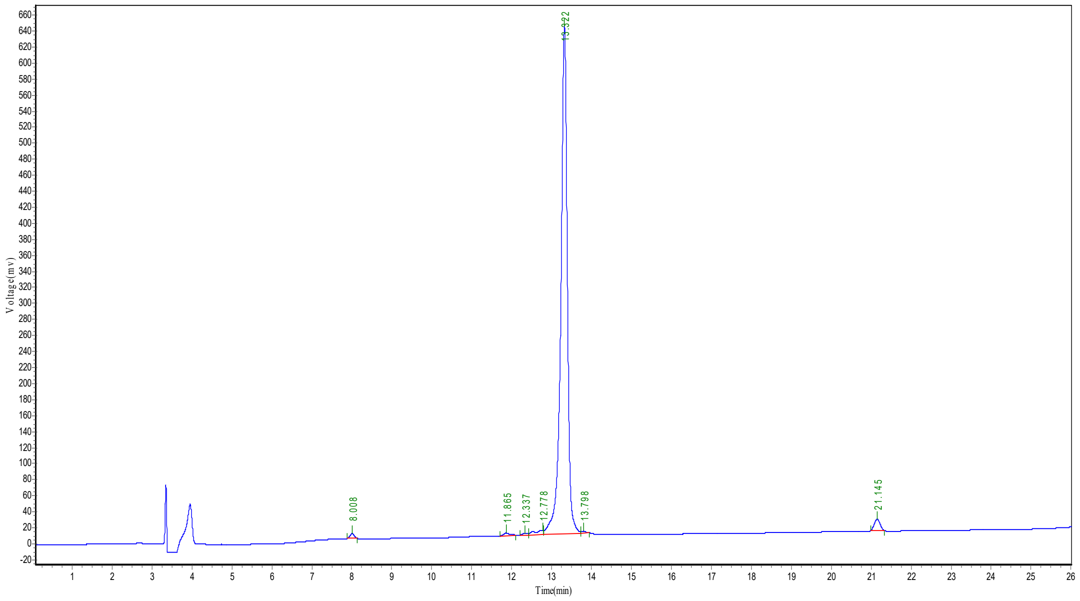

Peak No.	Ret Time	Height	Area	Conc.
1	8.008	6153.893	45897.758	0.6064
2	11.865	3507.283	36904.398	0.4876
3	12.337	2319.761	20712.061	0.2736
4	12.778	5217.545	82434.977	1.0891
5	13.322	633813.250	7223518.500	95.4367
6	13.798	2591.246	18004.016	0.2379
7	21.145	14353.593	141438.078	1.8687
Total:				100.0000

MS Spectrum

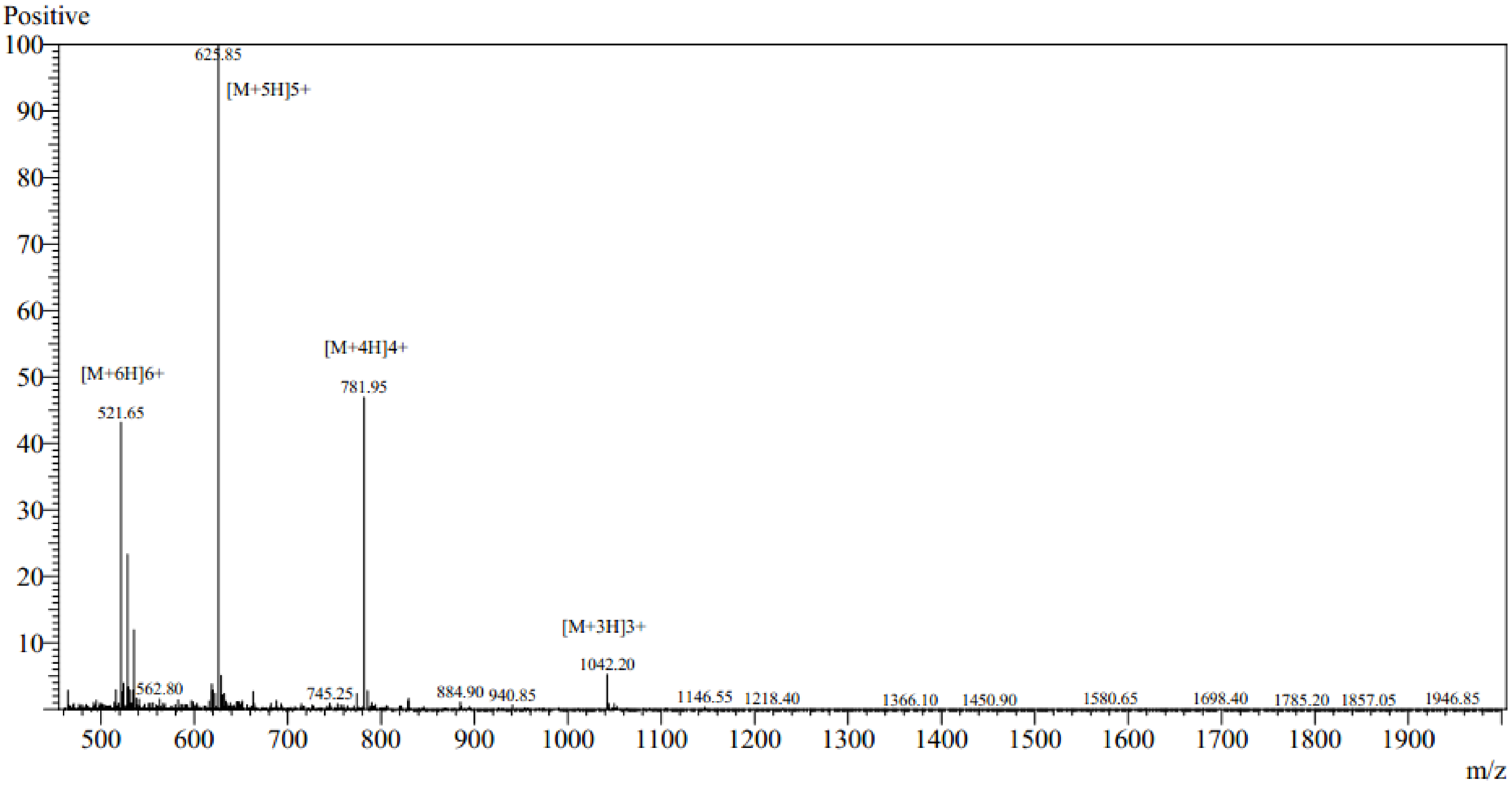

Acquired by	: Qiu	Probe	: ESI	Probe bias	: +4.5kv
Data Acquired	: 2018-12-15 15:43:34	Nebulizer Gas Floe	: 1.5L/min	Detector	: 2.0kv
Injection Volume	: 1	CDL	: -20.0v	T.Flow	: 0.2ml/min
Sample Name	: Lfcin DB FF-25(o)	CDL Temp	: 250℃	B.conc	: 50%H2O/50%ACN
Mw	: 3123.84	Block Temp	: 200℃		
Lot No.	: P151123-LR488159				

Lfcin C36G HPLC report

Product Name : Lfcin C36G FF-25-2

Instrument No. : 03019

Lot No. : P151123-LR488160

Column : 4.6*250mm, Kromasil C18 5um

Solvent A : 0.1% trifluoroacetic in 100% acetonitrile

Solvent B : 0.1% trifluoroacetic in 100% water

Gradient

	A	B
0.01min	19%	81%
25.0min	44%	56%
25.1min	100%	0%
30.0min	STOP	

Flow rate : 1.0ml/min

Wavelength : 220nm

Volume : 20ul

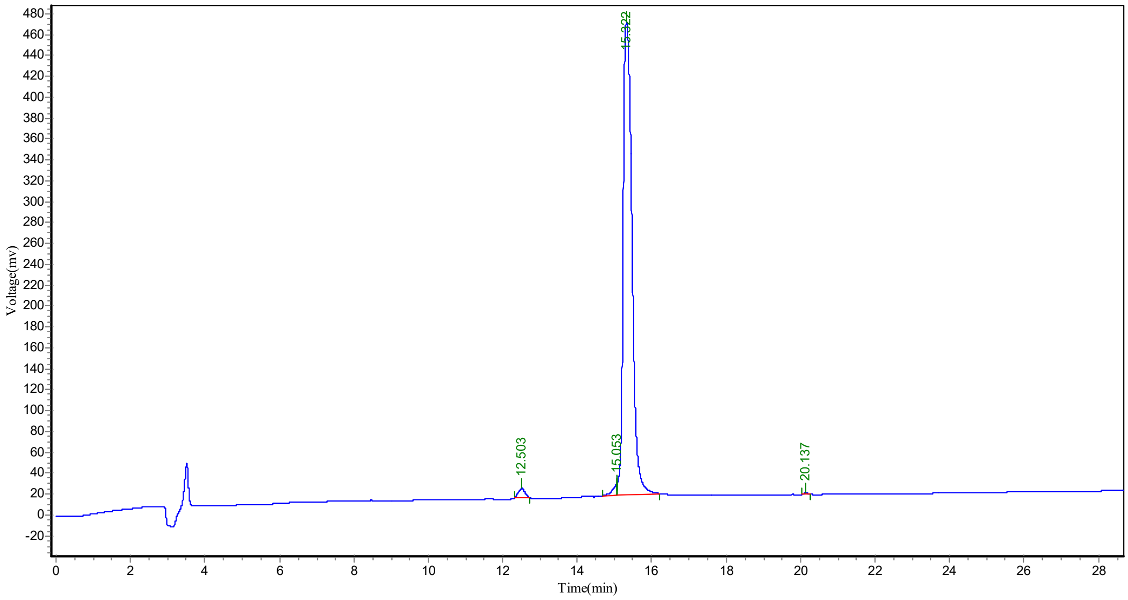

Rank	Time	Height	Area	Conc.
1	12.503	8915.875	109461.813	1.4362
2	15.053	9901.829	77006.805	1.0104
3	15.322	453179.906	7423755.500	97.4055
4	20.137	1486.578	11269.218	0.1479
Total				100

MS Spectrum

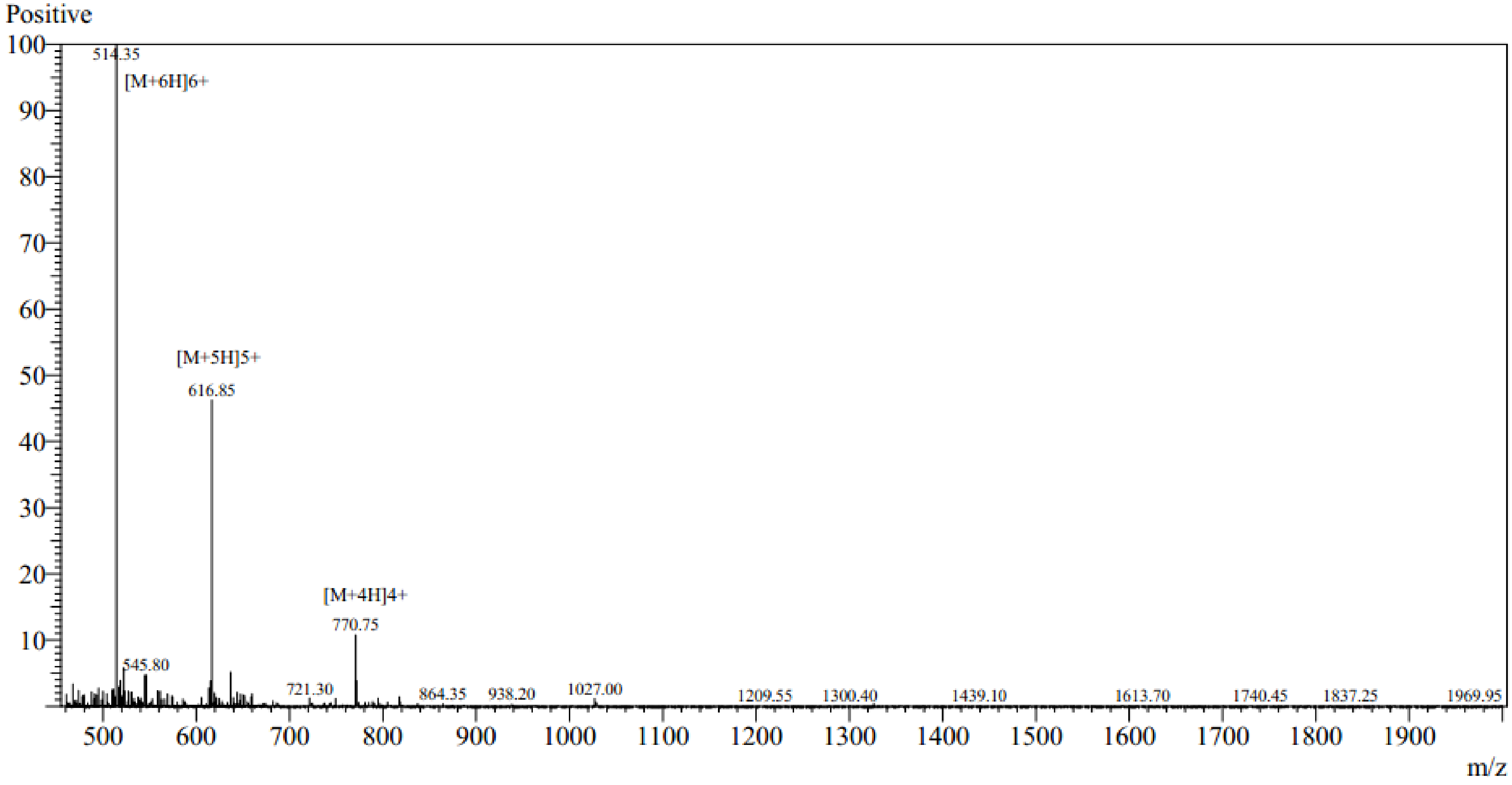

Acquired by	: Qiu	Probe	: ESI	Probe bias:	+4.5kv
Data Acquired	: 2018-12-16 11:55:24	Nebulizer Gas Floe:	1.5L/min	Detector	: 2.0kv
Injection Volume:	1	CDL	: -20.0v	T.Flow	: 0.2ml/min
Sample Name	: Lfcin C36G FF-25-2	CDL Temp	: 250℃	B.conc	: 50%H2O/50%ACN
Mw	: 3079.76	Block Temp	: 200℃		
Lot No.	: P151123-LR488160				
